# Leaf traits of prickly ash and its correlation with ecological and geographical factors of origin

**DOI:** 10.1038/s41598-024-56962-x

**Published:** 2024-03-15

**Authors:** Xixi Dong, Lin Shi, Shuqin Bao, Hao Fu, Yuming You, Yun Ren, Jichun Wang, Qiang Li, Zexiong Chen

**Affiliations:** 1 Chongqing Key Laboratory for Germplasm Innovation for Special Aromatic Spice Plants, Institute of Special Plants, College of Landscape Architecture and Life Science, Chongqing University of Arts and Sciences, Chongqing, 402160 China; 2 College of Agronomy and Biotechnology, Southwest University, Beibei, Chongqing, 400715 China; 3 Geological Team 607, Chongqing Geological and Mineral Exploration and Development Bureau, Chongqing, 401300 China

**Keywords:** Prickly ash, Origin, Latitude, Temperature, Leaf traits, Comprehensive evaluation, Ecology, Physiology, Plant sciences

## Abstract

The morphological, physiological, and biochemical characteristics of leaves result from the long-term adaptation of plants to their environment and are closely related to plant growth and development. In this study, 37 prickly ash germplasm resources from 18 production areas were utilized as the subjects of research. Logistic equations, principal component analysis, and cluster analysis were employed to comprehensively evaluate the leaf traits of prickly ash germplasm resources, with an analysis of their correlation with ecological and geographical factors in the production areas. The results showed that the leaf traits of prickly ash germplasms of different origins are substantially different and diverse. The coefficient of variation for the 14 leaf traits was greater than 10%. The coefficient of variation of the compound leaflet number was the highest among all the considered leaf traits, and the coefficient of variation of leaf thickness was the lowest, at 49.86% and 11.37%, respectively. The leaf traits of the prickly ash germplasm originating from Chongqing in Yongchuan, Chongqing in Rongchang, and Yunnan in Honghe ranked highest, whereas the leaf traits of the prickly ash germplasm from Henan in Jiaozuo, Gansu in Tianshui, and Shanxi in Yuncheng ranked lowest. The results of the correlation analysis showed that among the ecological and geographical factors of the origins, latitude had the strongest correlation with the leaf traits of the prickly ash germplasm. As latitude increased, the leaves of prickly ash gradually decreased in size, weight, and leaf shape index. The factor with the second strongest correlation was temperature. The leaves of the prickly ash germplasm originating from warmer climate areas were larger and heavier than those from areas with colder climates. Altitude and longitude did not significantly affect the leaf traits of the prickly ash germplasm, but at similar latitudes, the leaves of the prickly ash germplasm in high-altitude areas were smaller, and the leaves of the prickly ash germplasm in low-altitude areas were larger. These findings can provide valuable references for breeding and the sustainable utilization of new varieties of prickly ash resources.

## Introduction

Prickly ash, a small perennial tree belonging to the genus *Zanthoxylum* (Rutaceae), has a unique aromatic and pungent taste^1,2^. The leaf of Chinese prickly ash, a unique spice having typical pungent sensation, is a popular food in Southwest China with antipruritic, insecticidal and fungicidal functions^3^. This species is suitable for soil and water conservation^4,5^ and has therefore become one of the main tree species used for barren mountain management, ecological construction, poverty alleviation, and rural revitalization in China^6^. There are two species of *Zanthoxylum* in China, (1) red prickly ash (*Zanthoxylum bungeanum* Maxim.) and (2) green prickly ash (*Zanthoxylum armatum* DC.)^7^. Red prickly ash is mainly distributed in Gansu, Shaanxi, Shanxi, Henan, and Shandong provinces in the middle and lower reaches of the Yellow River^8^, whereas green prickly ash is mainly observed in Sichuan, Chongqing, Yunnan, Guizhou, and other locations in southwest China^9^.

Leaves have the largest contact area with the external environment and are most sensitive to environmental changes, which can directly reflect the adaptation of plants to the environment^10–12^. The temperature, altitude, and latitude of the origin of a plant can strongly impact the leaf traits of germplasm resources. The temperature of the origin affects the water and nutrient contents of the soil, which subsequently affects the morphological characteristics of the plant^13^. The plant leaves are significantly thicker in higher-temperature areas than leaves in lower-temperature areas^14,15^. The weight of plant leaves is significantly higher in higher-altitude areas compared with lower-altitude areas, suggesting an adaptation to improve mechanical resistance to the elements^16–18^. We studied the functional traits of *Cyclobalanopsis glauca* leaves at different altitudes on Wuyi Mountain. The results showed that leaf area and quality first increased and then decreased with increasing altitude. Tuo et al. analyzed the correlation between apparent leaf characteristics and altitude and showed that with increasing altitude, plant height significantly decreased^19^. Leaf and petiole lengths decreased with increasing altitude, whereas leaf width was less affected by altitude. In addition, an increase in altitude affects the intensity of solar radiation, and excessive radiation affects the chlorophyll a content in plants and hinders the formation of organelles^20,21^. Solar UV-B radiation destroys the membrane structure of chloroplasts, leading to disordered reactive oxygen metabolism and the degradation of photosynthetic pigments^22^. Mooney and Billings also showed that the chlorophyll content of plants in high-altitude areas is typically low^23^.

In summary, many studies have been conducted on the differences in leaf traits of plants. The leaf traits of plant species with different varieties from different origins can be considerably different. The leaf area of varieties originating from middle and low latitudes is larger than that of varieties originating from high latitudes. Notably, the correlation between the leaf traits of prickly ash germplasm resources and the ecological and geographical factors of origin has not been reported. Therefore, in this study, we assessed the effects of ecological and geographical factors on the leaf traits of prickly ash from 37 prickly ash germplasm resources from 18 origins as our study model. Principal component and cluster analyses were used to comprehensively evaluate the leaf traits of these prickly ash germplasm resources and to clarify the relationships of the leaf traits of prickly ash germplasm resources with the ecological and geographical factors of the origin to provide a theoretical basis and technical support for efficiently using prickly ash germplasm resources, breeding, and identifying the origin of new varieties.

## Results

### Differences in leaf characteristics of prickly ash germplasm resources from different origins

Significant differences were observed in petiole length, petiole width, leaf length, leaf width, leaf thickness, leaflet number, leaf area, and leaf shape index between the prickly ash germplasm resources from different origins (Table 1). In 2021, the average petiole length of 18 prickly ash germplasms was 5.43 cm, petiole width was 0.50 cm, leaf length was 6.94 cm, leaf width was 2.31 cm, leaf thickness was 0.02 mm, compound leaflet number was 6.30, leaf area was 12.62 cm^2^, and leaf type index was 2.83. Among them, the petiole of prickly ash in Tongchuan, Shaanxi, was the longest, reaching 7.63 cm and 40.52% longer than the average. The petiole of prickly ash in Ya'an, Sichuan, was the shortest, reaching 3.14 cm and 42.17% shorter than the average. The prickly ash leaf petiole from Honghe, Yunnan, was the widest, reaching 0.81 cm and 62.00% wider than the average, and the narrowest from Ya'an, Sichuan, reaching 0.28 cm and 44.00% narrower than the average. The longest and widest prickly ash leaves were from Yongchuan, Chongqing, being 8.91 cm and 3.03 cm, respectively. The shortest prickly ash leaf was from Wudu, at 5.78 cm, and the narrowest prickly ash leaf was from Yuncheng, Shanxi, at 1.78 cm. The thickest prickly ash leaves were observed in Yunnan Honghe, and the thinnest were from Gansu Tianshui, which were 0.02 mm and 0.01 mm, respectively. The number of compound leaves of prickly ash in Hancheng, Shaanxi, was the highest and lowest in Yongchuan, Chongqing, at 8.08% and 4.20%, respectively. The largest prickly ash leaf area was observed in Yunnan, Honghe, at 19.43 cm^2^, and the smallest was observed in Shanxi, Yuncheng, at only 7.94 cm^2^. The leaf shape index of the prickly ash in Kunming, Yunnan, was the largest and smallest in Jiaozuo, Henan, at 3.36 and 2.12, respectively.

**Table 1:** Leaf characteristics of prickly ash germplasm in 2021 and 2022.

Year	Area of origin	Petiole length /cm	Petiole width /cm	Leaf length /cm	Leaf width /cm	Thickleaf /mm	Number of compound leaflets	Leaf area /cm^2^	Leaf index
2021	Hancheng, Shanxi	6.21 ± 1.81 abcdefg	0.65 ± 0.21 abcde	6.18 ± 0.99 ab	2.14 ± 0.60 ab	0.02 ± 0.00 a	8.08 ± 1.29 ab	9.95 ± 3.56 defg	2.50 ± 1.13 abcdef
	Tongchuan, Shanxi	7.63 ± 1.50 a	0.51 ± 0.10 bcdefghij	6.47 ± 1.05 ab	2.12 ± 0.44 ab	0.01 ± 0.00 a	7.80 ± 1.00 abcd	10.39 ± 2.90 cdefg	2.30 ± 0.70 abcdef
	Tianshui, Gansu	6.64 ± 0.84 abcde	0.58 ± 0.13 bcdef	5.86 ± 0.80 b	2.00 ± 0.15 ab	0.01 ± 0.00 a	7.40 ± 0.84 abcdef	8.73 ± 0.86 fg	2.36 ± 0.53 abcdef
	Wudu, Gansu	6.70 ± 0.79 abcd	0.57 ± 0.13 bcdefgh	5.78 ± 0.78 b	2.10 ± 0.51 ab	0.01 ± 0.00 a	7.60 ± 0.97 abcde	9.27 ± 3.22 efg	2.26 ± 0.65 cdef
	Yuncheng, Shanxi	7.10 ± 1.12 ab	0.67 ± 0.09 abc	5.98 ± 1.11 b	1.78 ± 0.11 b	0.01 ± 0.00 a	7.20 ± 0.63 abcdefgh	7.94 ± 1.27 g	2.19 ± 0.87 ef
	Jiaozuo, Henan	6.70 ± 2.05 abcd	0.64 ± 0.14 abcde	5.94 ± 0.95 b	2.13 ± 0.64 ab	0.01 ± 0.00 a	7.40 ± 0.84 abcdef	9.68 ± 4.00 efg	2.12 ± 0.49 f.
	Asakura, Japan	6.29 ± 2.18 abcdefg	0.28 ± 0.05 k	6.97 ± 1.58 ab	2.61 ± 0.60 ab	0.02 ± 0.00 a	7.60 ± 1.31 abcde	11.25 ± 4.45 bcdefg	2.39 ± 0.62 abcdef
	Yaan, Sichuan	3.14 ± 1.01 h	0.28 ± 0.06 k	6.73 ± 1.31 ab	1.99 ± 0.44 ab	0.02 ± 0.00 a	7.27 ± 2.27 abcdefg	10.31 ± 3.64 cdefg	2.63 ± 0.92 abcdef
	Yingshan, Sichuan	4.27 ± 1.46 cdefgh	0.49 ± 0.11 bcdefghijk	6.59 ± 1.12 ab	2.42 ± 0.51 ab	0.02 ± 0.00 a	5.47 ± 0.86 defghi	12.30 ± 4.58 bcdefg	3.11 ± 0.48 abcdef
	Hongya, Sichuan	3.86 ± 1.10 efgh	0.52 ± 0.16 bcdefghij	6.84 ± 1.41 ab	2.15 ± 0.44 ab	0.02 ± 0.00 a	4.63 ± 0.56 i	14.94 ± 2.94 abcdef	3.20 ± 0.37 abcdef
	Zunyi, Guizhou	4.62 ± 1.51 bcdefgh	0.44 ± 0.08 efghijk	6.47 ± 1.03 ab	2.05 ± 0.43 ab	0.02 ± 0.00 a	5.67 ± 1.21 bcdefghi	9.97 ± 2.58 defg	3.32 ± 0.95 abc
	Rongchang, Chongqing	3.66 ± 1.40 fgh	0.32 ± 0.05 jk	8.40 ± 1.48 ab	2.63 ± 0.51 ab	0.02 ± 0.00 a	4.33 ± 0.96 i	16.84 ± 5.30 abc	3.28 ± 0.68 abcde
	Yongchuan, Chongqing	4.13 ± 0.86 defgh	0.34 ± 0.05 jk	8.91 ± 1.80 a	3.03 ± 0.23 a	0.02 ± 0.00 a	4.20 ± 1.03 i	17.30 ± 5.58 ab	3.33 ± 0.44 abc
	Jiangjin, Chongqing	3.26 ± 1.44 h	0.37 ± 0.11 fghijk	6.54 ± 1.35 ab	2.26 ± 0.53 ab	0.02 ± 0.00 a	4.84 ± 0.68 hi	13.69 ± 3.27 abcdefg	3.05 ± 0.80 abcdef
	Kunming, Yunnan	5.47 ± 1.40 abcdefgh	0.49 ± 0.08 becdfghijk	7.99 ± 1.47 ab	2.44 ± 0.46 ab	0.02 ± 0.00 a	5.40 ± 0.81 defghi	14.76 ± 4.20 abcdef	3.36 ± 0.81 a
	Yongshan, Yunnan	4.38 ± 1.92 bcdefgh	0.45 ± 0.12 cdefghijk	7.02 ± 0.94 ab	2.21 ± 0.53 ab	0.02 ± 0.00 a	5.98 ± 1.05 bcdefghi	14.88 ± 3.29 abcdef	3.33 ± 0.78 abc
	Honghe, Yunnan	7.50 ± 1.82 a	0.81 ± 0.19 a	8.45 ± 1.66 ab	3.00 ± 0.52 a	0.02 ± 0.00 a	6.40 ± 0.93 abcdefghi	19.43 ± 6.47 a	3.11 ± 0.35 abcdef
	Qujing, Yunnan	6.25 ± 2.04 abcdefg	0.67 ± 0.18 abcd	7.78 ± 1.88 ab	2.53 ± 0.67 ab	0.02 ± 0.00 a	6.20 ± 1.00 abcdefghi	15.51 ± 7.02 abc	3.13 ± 0.48 abcdef
2022	Hancheng, Shanxi	6.14 ± 0.69 abcdefg	0.64 ± 0.10 abcde	6.09 ± 0.83 ab	2.15 ± 0.61 ab	0.01 ± 0.00 a	8.09 ± 1.04 ab	9.99 ± 1.47 defg	2.46 ± 0.36 abcdef
	Tongchuan, Shanxi	7.59 ± 1.32 a	0.52 ± 0.07 bcdefghij	6.46 ± 0.95 ab	2.13 ± 0.52 ab	0.01 ± 0.01 a	7.91 ± 1.04 abc	10.43 ± 1.59 cdefg	2.27 ± 0.43 bcdef
	Tianshui, Gansu	6.45 ± 1.17 abcdef	0.57 ± 0.08 bcdefg	5.88 ± 0.98 b	2.01 ± 0.41 ab	0.01 ± 0.00 a	8.45 ± 0.93 a	8.86 ± 0.93 fg	2.25 ± 0.37 cdef
	Wudu, Gansu	6.75 ± 1.44 abcd	0.56 ± 0.08 bcdefghi	5.83 ± 0.66 b	2.16 ± 0.43 ab	0.01 ± 0.00 a	7.73 ± 1.35 abcde	9.39 ± 1.01 efg	2.35 ± 0.38 abcdef
	Yuncheng, Shanxi	6.99 ± 1.41 abc	0.67 ± 0.11 abcd	5.97 ± 0.81 b	1.79 ± 0.63 b	0.02 ± 0.00 a	7.36 ± 1.50 abcdef	9.28 ± 1.04 efg	2.21 ± 0.43 def
	Jiaozuo, Henan	6.87 ± 1.36 abcd	0.65 ± 0.13 abcde	5.90 ± 0.80 b	2.10 ± 0.56 ab	0.01 ± 0.00 a	7.55 ± 1.57 abcde	8.01 ± 0.86 g	2.16 ± 0.47 f.
	Asakura, Japan	6.25 ± 0.81 abcdefg	0.36 ± 0.06 fghijk	6.25 ± 0.59 ab	2.24 ± 0.47 ab	0.01 ± 0.00 a	7.18 ± 1.66 abcdefgh	11.16 ± 1.75 bcdefg	2.37 ± 0.64 abcdef
	Yaan, Sichuan	3.12 ± 0.61 h	0.37 ± 0.07 fghijk	6.44 ± 0.82 ab	2.19 ± 0.40 ab	0.01 ± 0.00 a	7.55 ± 1.81 abcde	10.53 ± 1.38 cdefg	2.69 ± 0.39 abcdef
	Yingshan, Sichuan	4.13 ± 1.01 defgh	0.49 ± 0.08 bcdefghijk	6.46 ± 0.77 ab	2.54 ± 0.34 ab	0.02 ± 0.00 a	5.55 ± 0.93 cdefghi	14.49 ± 2.90 abcdefg	3.10 ± 0.42 abcdef
	Hongya, Sichuan	3.74 ± 0.84 fgh	0.51 ± 0.09 bcdefghij	6.74 ± 1.02 ab	2.70 ± 0.56 ab	0.02 ± 0.00 a	4.91 ± 0.83 ghi	15.18 ± 2.70 abcdef	3.17 ± 0.45 abcdef
	Zunyi, Guizhou	4.60 ± 1.22 bcdefgh	0.45 ± 0.09 efghijk	6.43 ± 1.10 ab	2.03 ± 0.66 ab	0.02 ± 0.00 a	6.09 ± 1.04 abcdefghi	9.88 ± 3.62 efg	3.10 ± 0.64 abcdef
	Rongchang, Chongqing	3.61 ± 1.07 gh	0.35 ± 0.08 ghijk	8.09 ± 2.19 ab	2.92 ± 0.60 ab	0.02 ± 0.00 a	4.27 ± 1.01 i	15.91 ± 4.08 abcde	3.28 ± 0.43 abcd
	Yongchuan, Chongqing	4.20 ± 0.69 cdefgh	0.35 ± 0.12 ijk	8.26 ± 2.11 ab	3.02 ± 0.69 a	0.02 ± 0.00 a	4.27 ± 1.01 i	16.95 ± 3.11 abc	3.35 ± 0.47 ab
	Jiangjin, Chongqing	3.22 ± 1.16 h	0.35 ± 0.10 hijk	6.83 ± 0.69 ab	2.50 ± 0.56 ab	0.02 ± 0.00 a	5.00 ± 0.00 fghi	14.37 ± 2.94 abcdefg	3.02 ± 0.34 abcdef
	Kunming, Yunnan	5.11 ± 1.39 abcdefgh	0.48 ± 0.07 bcdefghijk	7.66 ± 2.18 ab	2.49 ± 0.29 ab	0.02 ± 0.00 a	5.36 ± 0.81 efghi	14.61 ± 4.17 abcdefg	3.34 ± 0.38 abc
	Yongshan, Yunnan	4.55 ± 0.94 bcdefgh	0.45 ± 0.07 defghijk	7.07 ± 2.11 ab	2.39 ± 0.49 ab	0.02 ± 0.00 a	5.09 ± 0.70 fghi	14.85 ± 3.64 abcdef	3.20 ± 0.35 abcdef
	Honghe, Yunnan	7.53 ± 1.10 a	0.67 ± 0.13 ab	8.24 ± 1.82 ab	2.88 ± 0.72 ab	0.02 ± 0.00 a	6.27 ± 1.01 abcdefghi	16.65 ± 2.99 abcd	3.17 ± 0.50 abcdef
	Qujing, Yunnan	6.11 ± 1.32 abcdefg	0.66 ± 0.14 abced	7.59 ± 1.30 ab	2.50 ± 0.74 ab	0.02 ± 0.00 a	6.09 ± 1.04 abcdefghi	14.79 ± 2.79 abcdef	3.11 ± 0.47 abcdef
F value	Years (Y)	19.98**	0.01^ ns^	1.44^ ns^	0.31^ ns^	0.71^ ns^	4.34*	0.01^ ns^	0.00^ ns^
	Area of origin (A)	18.83**	30.67**	6.40**	5.42**	5.14**	25.15**	17.79**	15.08**
	Y × A	3.64**	1.47^ ns^	2.07**	2.66**	0.48^ ns^	1.47^ ns^	2.85**	0.58^ ns^

Different lowercase letters in the same column represent a significant difference (*p* < 0.05); ** indicates the difference was extremely significant at the 1% level (*p* < 0.01); * indicates the difference was significant at the 5% level (*p* < 0.05); ns indicates that the difference or relationship being tested is not statistically significant.

In 2022, the overall average petiole length of prickly ash from all origins was 5.39 cm, petiole width was 0.51 cm, leaf length was 6.79 cm, leaf width was 2.37 cm, leaf thickness was 0.02 mm, compound leaflet number was 6.37, leaf area was 12.52 cm^2^, and leaf type index was 2.81. The petiole of the prickly ash in Tongchuan, Shaanxi, was the longest, reaching 7.59 cm and 40.82% longer than the average petiole length. The petiole of prickly ash in Ya'an, Sichuan, was the shortest, at 3.12 cm and 72.76% of the average petiole length. The widest petiole of prickly ash was observed in Yunnan, Honghe, at 0.67 cm, which was 31.37% wider than the average petiole width, and the narrowest was observed in Japan, Chaocang, at 0.36 cm and 29.41% narrower than the average petiole width. The longest and widest prickly ash leaves from Yongchuan, Chongqing were 8.26 cm and 3.02 cm, respectively. The shortest leaf was observed in Wudu, Gansu, and the narrowest leaf width was from Yuncheng, Shanxi, at 5.83 cm and 1.79 cm, respectively. The thickest leaves were from Yongshan, Yunnan, and the thinnest from Tianshui, Gansu, at 0.02 and 0.01 mm, respectively. The prickly ash in Hancheng, Shaanxi, had the highest number of compound leaves, and those in Yongchuan, Chongqing, had the smallest number, at 8.09 and 4.27, respectively. The largest prickly ash leaf shape index of 16.65 cm^2^ was observed in Yunnan, Honghe, and the smallest was observed in Jiaozuo, at 8.01 cm^2^. The largest prickly ash leaf shape index was observed in Kunming, Yunnan, and the smallest was recorded in Jiaozuo, Henan, at 3.34 and 2.16, respectively.

### Differences in leaf quality and chlorophyll content of prickly ash from different origins

Significant differences were observed in chlorophyll a, chlorophyll b, total chlorophyll, and carotenoid contents, and fresh and dry weights of the prickly ash leaf germplasm of different origins (Table 2). In 2021, the average chlorophyll a, chlorophyll b, total chlorophyll, and carotenoid contents were 0.83, 0.24, 1.08, and 0.27 mg/g, respectively; the average fresh and dry leaf weights were 0.31 g and 0.14 g, respectively. The highest chlorophyll a content (1.20 mg/g) was measured in the prickly ash in Yongchuan, Chongqing, at 44.58% higher content than the average. The lowest chlorophyll a content was measured in prickly ash in Qujing, Yunnan, at 0.35 mg/g, which was 57.83% lower than the average. The highest and lowest chlorophyll b contents were measured in Shaanxi, Tongchuan, and Yunnan, Qujing at 0.36 mg/g and 0.12 mg/g, respectively. The total chlorophyll content was the highest in Yongchuan, Chongqing, and the lowest in Qujing, Yunnan, at 1.55 mg/g and 0.47 mg/g, respectively. The carotenoid content of the prickly ash leaves in Yongchuan, Chongqing, was the highest, at 0.34 mg/g, and lowest in Qujing, Yunnan, at 0.17 mg/g. The fresh and dry weights of the leaves were the highest in Honghe, Yunnan, at 0.37 g and 0.19 g, respectively, and the lowest was in Tongchuan, Shaanxi, and Yingshan, Sichuan, at 0.20 g and 0.10 g, respectively.

**Table 2:** Pigment characteristics of prickly ash germplasm leaves in 2021 and 2022.

Year	Origin	Chlorophyll a / (mg/g)	Chlorophyll b/(mg/g)	Total chlorophyll /(mg/g)	Carotenoids /(mg/g)	Fresh weight /g	Dry weight /g
2021	Hancheng, Shanxi	0.99 ± 0.05 abc	0.17 ± 0.09ghij	1.17 ± 0.09bcdefg	0.25 ± 0.12abcdef	0.25 ± 0.09cde	0.13 ± 0.02efghi
	Tongchuan, Shanxi	0.99 ± 0.03 abcd	0.36 ± 0.05a	1.35 ± 0.06ab	0.33 ± 0.01ab	0.20 ± 0.09e	0.14 ± 0.01defghi
	Tianshui, Gansu	0.97 ± 0.04 abcd	0.28 ± 0.05abcdefg	1.26 ± 0.07abcde	0.28 ± 0.02abcde	0.23 ± 0.04de	0.14 ± 0.01defgh
	Wudu, Gansu	0.98 ± 0.04 abcd	0.30 ± 0.03abcdef	1.28 ± 0.03abcd	0.29 ± 0.02abcde	0.26 ± 0.07bcde	014 ± 0.01defgh
	Yuncheng, Shanxi	1.00 ± 0.03 abc	0.29 ± 0.02abcdefg	1.29 ± 0.04abc	0.29 ± 0.03abcde	0.25 ± 0.07cde	0.14 ± 0.01defghi
	Jiaozuo, Henan	1.02 ± 0.04 abc	0.31 ± 0.07abcde	1.33 ± 0.06abc	0.28 ± 0.05abcde	0.26 ± 0.11bcde	0.14 ± 0.00defghi
	Asakura, Japan	0.86 ± 0.22 bcde	0.24 ± 0.06abcdefghi	1.11 ± 0.28bcdefg	0.31 ± 0.05abcd	0.34 ± 0.13abcde	0.13 ± 0.01defghi
	Yaan, Sichuan	0.69 ± 0.10 efgh	0.20 ± 0.03defghij	0.89 ± 0.13fghij	0.25 ± 0.02abcdef	0.29 ± 0.12abcde	0.14 ± 0.02defghi
	Yingshan, Sichuan	0.76 ± 0.06 cdef	0.20 ± 0.01cdefghij	0.95 ± 0.07defghi	0.21 ± 0.04bcdef	0.31 ± 0.09abcde	0.10 ± 0.00i
	Hongya, Sichuan	0.68 ± 0.04 efgh	0.18 ± 0.00efghij	0.86 ± 0.04ghij	0.24 ± 0.01abcdef	0.33 ± 0.11abcde	0.13 ± 0.01efghi
	Zunyi, Guizhou	1.02 ± 0.04 ab	0.31 ± 0.02abcde	1.33 ± 0.06abc	0.32 ± 0.04abcd	0.30 ± 0.08abcde	0.14 ± 0.00defgh
	Rongchang, Chongqing	0.90 ± 0.08 bcde	0.29 ± 0.06abcdefg	1.18 ± 0.13bcdefg	0.31 ± 0.05abcd	0.38 ± 0.13abcd	0.15 ± 0.03cdef
	Yongchuan, Chongqing	1.20 ± 0.00 a	0.35 ± 0.00ab	1.55 ± 0.00a	0.34 ± 0.00a	0.42 ± 0.11abc	0.14 ± 0.01defgh
	Jiangjin, Chongqing	0.79 ± 0.23 bcde	0.22 ± 0.05cdefghij	1.01 ± 0.27cdefgh	0.26 ± 0.04abcdef	0.31 ± 0.08abcde	0.12 ± 0.01ghi
	Kunming, Yunnan	0.44 ± 0.06 hij	0.15 ± 0.02hij	0.59 ± 0.06jk	0.21 ± 0.03cdef	0.37 ± 0.16abcde	0.18 ± 0.02abc
	Yongshan, Yunnan	0.88 ± 0.15 bcde	0.24 ± 0.05abcdefghij	1.12 ± 0.19bcdefg	0.26 ± 0.04abcdef	0.31 ± 0.07abcde	0.12 ± 0.01ghi
	Honghe, Yunnan	0.50 ± 0.02 ghij	0.17 ± 0.01ghij	0.67 ± 0.02ijk	0.23 ± 0.01abcdef	0.47 ± 0.16a	0.19 ± 0.02ab
	Qujing, Yunnan	0.35 ± 0.03 j	0.12 ± 0.01j	0.47 ± 0.04 k	0.17 ± 0.01ef	0.35 ± 0.14abcde	0.16 ± 0.01cdef
2022	Hancheng, Shanxi	0.98 ± 0.06 abcd	0.19 ± 0.04defghij	1.17 ± 0.07bcdefg	0.25 ± 0.02abcdef	0.26 ± 0.07bcde	0.13 ± 0.01defghi
	Tongchuan, Shanxi	1.01 ± 0.03 abc	0.33 ± 0.03abc	1.34 ± 0.02abc	0.33 ± 0.02abc	0.20 ± 0.05e	0.13 ± 0.01efghi
	Tianshui, Gansu	0.99 ± 0.05 abcd	0.27 ± 0.02abcdefgh	1.26 ± 0.07abcde	0.28 ± 0.03abcde	0.23 ± 0.04de	0.14 ± 0.01defghi
	Wudu, Gansu	0.98 ± 0.01 abcd	0.29 ± 0.02abcdefg	1.27 ± 0.03abcde	0.29 ± 0.02abcde	0.24 ± 0.04cde	0.13 ± 0.01defghi
	Yuncheng, Shanxi	0.99 ± 0.02 abc	0.28 ± 0.01abcdefg	1.27 ± 0.02abcde	0.28 ± 0.02abcdef	0.25 ± 0.04cde	0.14 ± 0.01defghi
	Jiaozuo, Henan	0.98 ± 0.03 abcd	0.31 ± 0.03abcd	1.30 ± 0.06abc	0.29 ± 0.03abcde	0.25 ± 0.04cde	0.14 ± 0.01defghi
	Asakura, Japan	0.85 ± 0.03 bcde	0.22 ± 0.04cdefghij	1.06 ± 0.06bcdefg	0.29 ± 0.02abcde	0.35 ± 0.13abcde	0.13 ± 0.01defghi
	Yaan, Sichuan	0.67 ± 0.02 efghi	0.20 ± 0.03defghij	0.87 ± 0.05fghij	0.25 ± 0.03abcdef	0.30 ± 0.08abcde	0.13 ± 0.01defghi
	Yingshan, Sichuan	0.73 ± 0.05 defg	0.21 ± 0.02cdefghij	0.94 ± 0.07efghi	0.21 ± 0.03def	0.30 ± 0.07abcde	0.11 ± 0.01hi
	Hongya, Sichuan	0.69 ± 0.04 efgh	0.19 ± 0.02defghij	0.88 ± 0.05fghij	0.23 ± 0.02abcdef	0.31 ± 0.05abcde	0.12 ± 0.01efghi
	Zunyi, Guizhou	1.01 ± 0.02 abc	0.31 ± 0.02abcde	1.32 ± 0.00abc	0.31 ± 0.02abcd	0.29 ± 0.10abcde	0.14 ± 0.00defgh
	Rongchang, Chongqing	0.91 ± 0.02 bcde	0.29 ± 0.02abcdefg	1.20 ± 0.03bcdef	0.30 ± 0.02abcd	0.31 ± 0.04abcde	0.16 ± 0.02bcde
	Yongchuan, Chongqing	1.18 ± 0.05 a	0.35 ± 0.01ab	1.53 ± 0.04a	0.33 ± 0.02abc	0.32 ± 0.05abcde	0.15 ± 0.01defg
	Jiangjin, Chongqing	0.82 ± 0.04 bcde	0.21 ± 0.04cdefghij	1.03 ± 0.05bcdefg	0.25 ± 0.01abcdef	0.29 ± 0.05abcde	0.12 ± 0.01fghi
	Kunming, Yunnan	0.42 ± 0.04 ij	0.14 ± 0.01ij	0.56 ± 0.05jk	0.22 ± 0.03abcdef	0.32 ± 0.09abcde	0.19 ± 0.01a
	Yongshan, Yunnan	0.91 ± 0.03 bcde	0.23 ± 0.02bcdefghij	1.14 ± 0.05bcdefg	0.27 ± 0.02abcdef	0.30 ± 0.05abcde	0.12 ± 0.01efghi
	Honghe, Yunnan	0.51 ± 0.02 fghij	0.18 ± 0.02fghij	0.69 ± 0.03hijk	0.24 ± 0.01abcdef	0.44 ± 0.15ab	0.18 ± 0.01abc
	Qujing, Yunnan	0.32 ± 0.01 j	0.13 ± 0.02ij	0.45 ± 0.01 k	0.16 ± 0.02f.	0.33 ± 0.08abcde	0.16 ± 0.01abcd
F value	Years (Y)	11.93**	1.06^ ns^	5.46*	0.19^ ns^	9.45**	24.70**
	Area of origin (A)	37.56**	11.59**	35.99**	5.36**	7.08**	13.85**
	Y × A	12.89**	8.03**	13.16**	3.20**	1.41^ ns^	8.89**

Different lowercase letters in the same column represent a significant difference (*p* < 0.05); ** indicates the difference was extremely significant at the 1% level (*p* < 0.01); * indicates the difference was significant at the 5% level (*p* < 0.05); ns indicates that the difference or relationship being tested is not statistically significant.

In 2022, the average chlorophyll a, chlorophyll b, total chlorophyll, and carotenoid contents were 0.83, 0.24, 1.07, and 0.26 mg/g, respectively; the average fresh and dry leaf weights were 0.29 g and 0.14 g. The highest chlorophyll a content was recorded in Yongchuan, Chongqing, at 1.18 mg/g, which was 42.17% higher than the average, and the lowest was measured in Qujing, Yunnan, at 0.32 mg/g, which was 61.45% lower than the average. The highest and lowest chlorophyll b contents were 0.33 mg/g and 0.13 mg/g in Tongchuan, Shaanxi, and Qujing, Yunnan, respectively. The total chlorophyll content was the highest in Yongchuan, Chongqing, and the lowest in Qujing, Yunnan, at 1.53 mg/g and 0.45 mg/g, respectively. The carotenoid content of the prickly ash leaves in Yongchuan, Chongqing, was also the highest, at 0.33 mg/g, and the lowest was measured in Qujing, Yunnan, at only 0.16 mg/g. The fresh weight of the leaves was the highest in Honghe, Yunnan, at 0.44 g, and the lowest in Tongchuan, Shaanxi, at 0.20 g. The dry weight of leaves was the highest in Kunming, Yunnan, and the lowest in Yingshan, Sichuan, at 0.19 g and 0.11 g, respectively.

### Variations in prickly ash leaf traits

The statistical results of the diversity of the 37 prickly ash germplasm resources from 18 origins after 2 years of average data for 14 leaf traits are shown in Table 3. The coefficient of variation for each index ranged from 11.37–49.86%, and the average coefficient of variation for each trait was 22.70%. The range of variation was wide, and the diversity of the resources was high. The range of the compound leaflet number was the largest, being 4.24–8.09, and the coefficient of variation reached as high as 49.86%. The range in the leaf thickness was the narrowest, at 0.01–0.02 mm, and the coefficient of variation was 11.37%.

**Table 3:** Statistical analysis of the diversity of prickly ash germplasm leaf traits from different origins.

Traits	Average value	Maximum	Minimum	Extreme difference	Standard deviation	Variable coefficient/%
Petiole length	5.41	7.61	3.13	4.48	1.51	27.99
Petiole width	0.50	0.74	0.32	0.42	0.13	26.44
Leaf length	6.86	8.59	5.81	2.78	0.90	13.06
Leaf width	2.34	3.02	1.79	1.24	0.33	14.07
Leaf thickness	0.02	0.02	0.01	0.01	0.00	11.37
Number of compound leaflets	6.34	8.09	4.24	3.85	3.16	49.86
Leaf area	12.57	18.04	8.61	9.44	3.16	25.15
Leaf index	2.82	3.35	2.14	1.21	0.46	16.25
Fresh weight	0.30	0.45	0.20	0.26	0.06	19.68
Dry weight	0.14	0.18	0.11	0.08	0.02	14.23
Chlorophyll a	0.83	1.19	0.34	0.85	0.23	27.56
Chlorophyll b	0.24	0.35	0.12	0.23	0.07	28.31
Total chlorophyll	1.07	1.54	0.46	1.08	0.29	27.12
Carotenoids	0.27	0.33	0.17	0.17	0.04	16.72

The average petiole length and width were 5.41 and 0.50 cm, respectively, and the coefficients of variation were 27.99% and 26.44%, respectively. The longest petiole was recorded in Tongchuan, Shaanxi (7.61 cm), and the shortest in Ya'an, Sichuan (3.13 cm). The widest petiole was observed in Yunnan, Honghe (0.74 cm), and the narrowest in Japan, Chaocang (0.32 cm). The average leaf length, width, thickness, and number of compound leaflets were 6.86 cm, 2.34 cm, 0.02 mm, and 6.34, respectively. The coefficients of variation were 13.06%, 14.07%, 11.37%, and 49.86%, respectively. The average values of leaf area, leaf type index, leaf fresh weight, and leaf dry weight were 12.57 cm^2^, 2.82, 0.30 g, and 0.14 g, respectively; the coefficients of variation were 25.15%, 16.25%, 19.68%, and 14.23%, respectively.

Variations were also identified in the pigment content of the prickly ash leaves. The average contents of chlorophyll a, chlorophyll b, total chlorophyll, and carotenoid were 0.83, 0.24, 1.07, and 0.27 mg/g, respectively, and the coefficients of variation were 27.56%, 28.31%, 27.12%, and 16.72%, respectively. The contents of chlorophyll a, chlorophyll b, total chlorophyll, and carotenoid were highest in Yongchuan, Chongqing, at 1.19, 0.35, 1.54, and 0.33 mg/g, and lowest in Qujing, Yunnan, at 0.34, 0.12, 0.46, and 0.17 mg/g.

### Correlation of eco-geographical factors and prickly ash leaf traits

**Figure 1:**
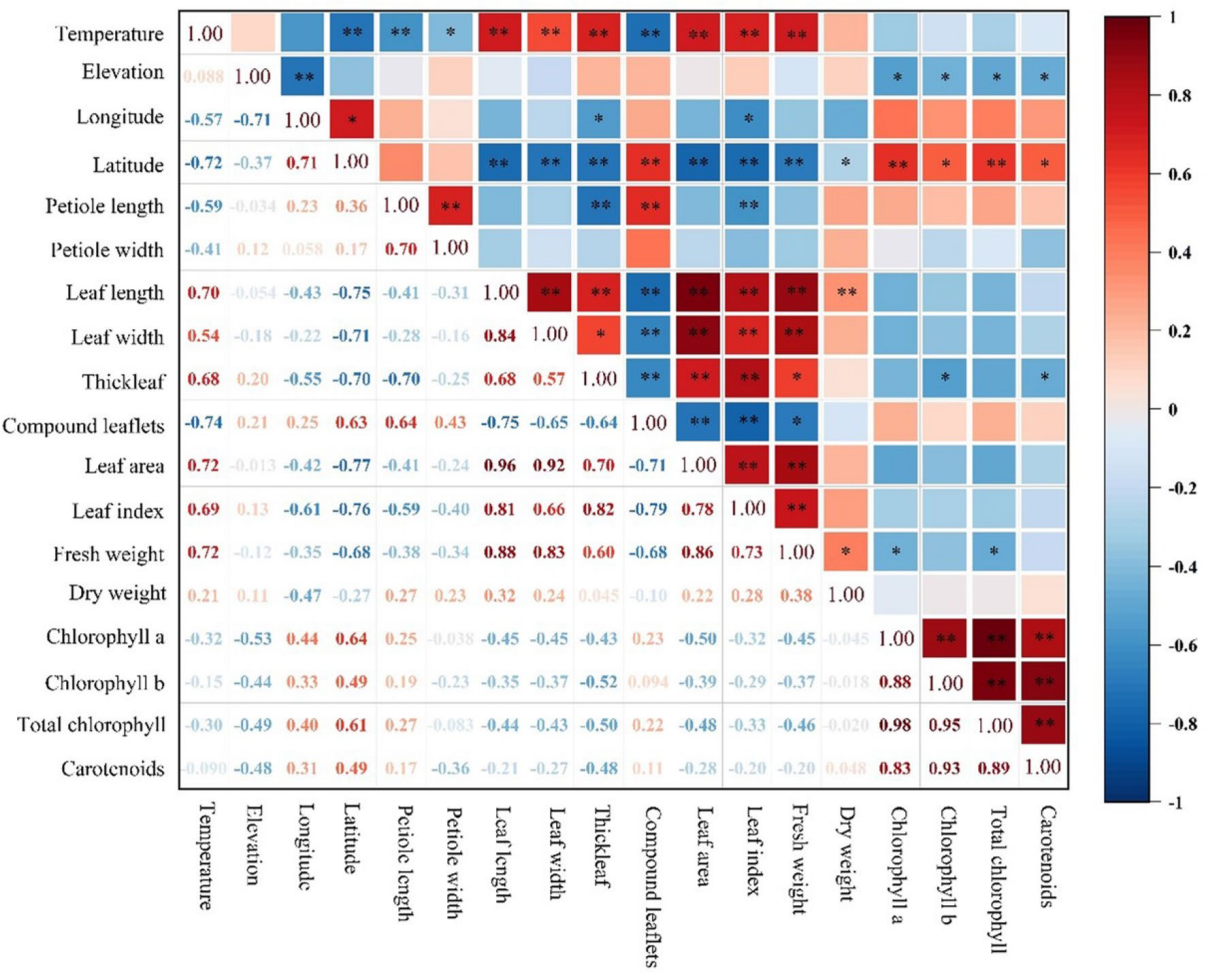
Correlation analysis between leaf traits and ecological and geographical factors of prickly ash in each area of origin. “*” indicates a significant difference at *p* < 0.05, “**” indicates an extremely significant difference at *p* < 0.01.

The results in Fig. 1 show that the ecological and geographical factors of the place of origin strongly influenced the leaf traits of prickly ash. A significant correlation was identified between the temperature of the origin and multiple leaf traits. The temperature was significantly positively correlated with leaf length (*R*^2^ = 0.70 **), leaf width (*R*^2^ = 0.54 **), leaf thickness (*R*^2^ = 0.68 **), leaf area (*R*^2^ = 0.72 **), leaf shape index (*R*^2^ = 0.69 **), and fresh leaf weight (*R*^2^ = 0.72 **), and significantly negatively correlated with petiole length (*R*^2^ = − 0.59 **) and compound leaflet number (*R*^2^ = − 0.74 **). The altitude of the variety origin was significantly correlated with the photosynthetic pigment content of prickly ash leaves and significantly negatively correlated with chlorophyll a (*R*^2^ = − 0.53 *), chlorophyll b (*R*^2^ = − 0.44 *), total chlorophyll (*R*^2^ = − 0.49 *), and carotenoid (*R*^2^ = − 0.48 *) contents. The longitude of the origin was significantly negatively correlated with leaf thickness (*R*^2^ = − 0.55 *) and leaf shape index (*R*^2^ = − 0.61 *). The latitude of origin was significantly positively correlated with the number of compound leaves (*R*^2^ = 0.63 **) and chlorophyll a (*R*^2^ = 0.64 **) and total chlorophyll (*R*^2^ = 0.61 **) contents and significantly negatively correlated with leaf length (*R*^2^ = − 0.75), leaf width (*R*^2^ = − 0.71 **), leaf thickness (*R*^2^ = − 0.70 **), leaf area (*R*^2^ = − 0.77 **), leaf shape index (*R*^2^ = − 0.76 **), and fresh leaf weight (*R*^2^ = − 0.68 **).

Further analysis showed a significant correlation between some leaf traits in the prickly ash plants. Petiole length was significantly positively correlated with petiole width and compound leaflet number and significantly negatively correlated with leaf thickness and the leaf shape index. Leaf length was significantly positively correlated with leaf thickness, leaf area, leaf type index, leaf fresh weight, and dry leaf weight and significantly negatively correlated with the number of compound leaves. Leaf width was significantly positively correlated with leaf area, leaf shape index, and leaf fresh weight and significantly negatively correlated with the number of compound leaflets. Leaf thickness was significantly positively correlated with leaf area and leaf type index and significantly negatively correlated with the number of compound leaflets, chlorophyll b, and carotenoid contents. The number of compound leaflets was significantly negatively correlated with the leaf area and leaf type index. Leaf area was significantly positively correlated with the leaf type index and leaf area. The leaf area, leaf type index, and fresh leaf weight were significantly and positively correlated with each other. The contents of the four photosynthetic pigments in the leaves were also significantly and positively correlated with each other.

### Cluster analysis based on prickly ash leaf traits

**Figure 2:**
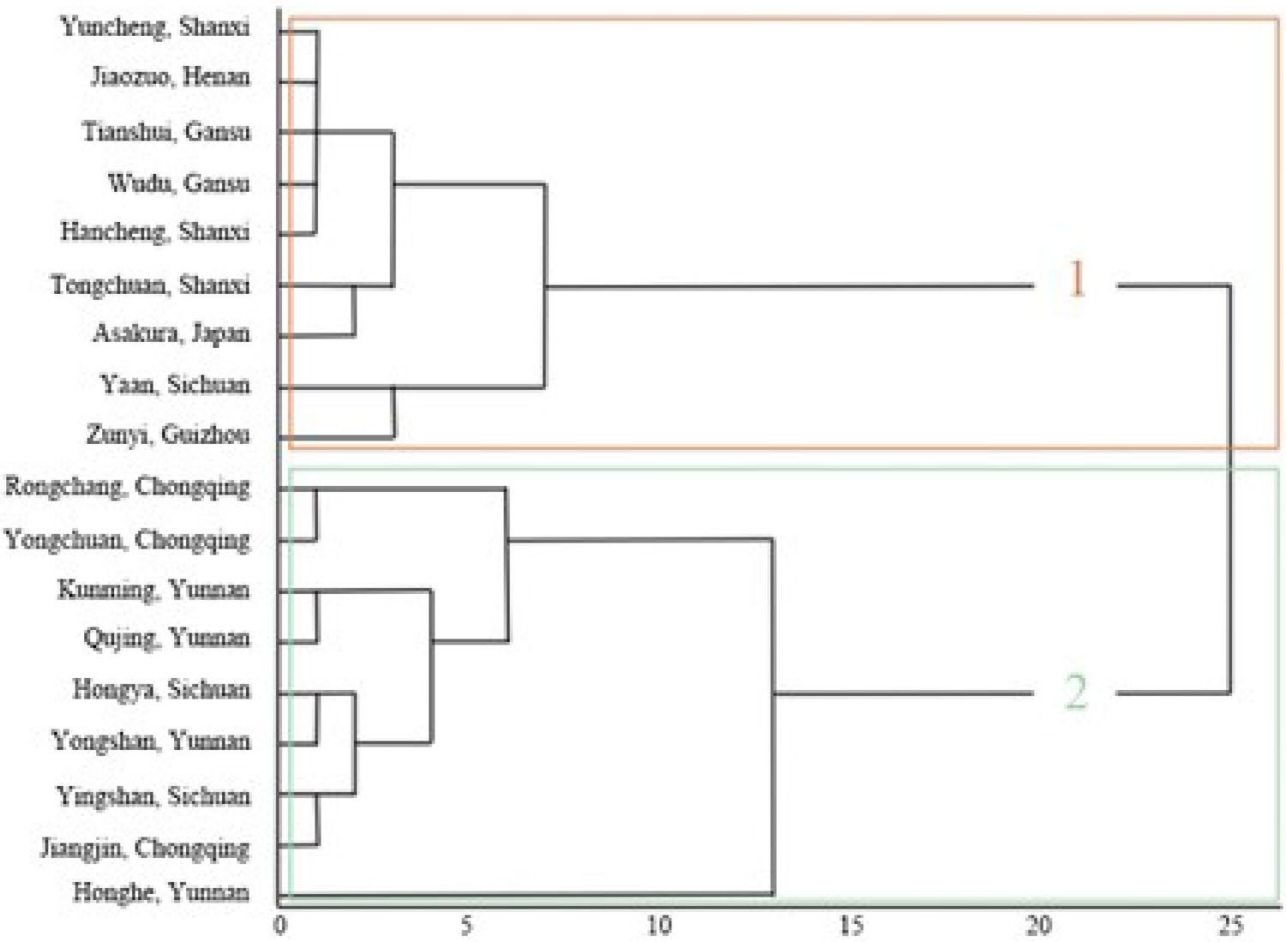
Cluster analysis based on leaf traits of prickly ash.

Cluster analysis of 37 prickly ash germplasm resources from 18 origins was performed using the leaf traits of prickly ash. The results indicated that they could be divided into two groups at 15 Euclidean distances (Fig. 2). The first category included 17 prickly ash germplasms from 9 regions, including Hancheng, Tongchuan, and Tianshui. All of them were red except for the small prickly ash from Ya'an, Sichuan. The average temperature, altitude, longitude, and latitude of these nine origins were − 9.56 °C, 1383.89 m, 110.25°E, and 33.59°N, respectively. The average petiole length and width of the 17 germplasms were 6.10 cm and 0.52 cm, respectively. The average leaf length, width, thickness, and compound leaflet number were 6.20 cm, 2.09 cm, 0.01 mm, and 7.44, respectively. The average leaf area and leaf type index were 9.72 cm^2^ and 2.44, respectively. The average fresh and dry leaf weights were 0.26 g and 0.14 g, respectively. The average chlorophyll a, chlorophyll b, total chlorophyll, and carotenoid contents were 0.94, 0.27, 1.21, and 0.29 mg/g, respectively.

The second category included 20 prickly ash germplasms from 9 origins, including Yingshan in Sichuan, Hongya in Sichuan, and Rongchang in Chongqing. The average temperature, altitude, longitude, and latitude of the nine origins were − 2.00 °C, 1322.89 m, 104.89°E, and 27.65°N, respectively. The average petiole length and width of the 20 prickly ash germplasm leaves were 4.72 cm and 0.49 cm, respectively. The average leaf length, width, thickness, and compound leaflet number were 7.53 cm, 2.59 cm, 0.02 mm, and 5.24, respectively. The average leaf area and leaf type index were 15.41 cm^2^ and 3.20, respectively. The average fresh and dry leaf weights were 0.34 g and 0.14 g, respectively. The chlorophyll a, chlorophyll b, total chlorophyll, and carotenoid contents were 0.72, 0.21, 0.93, and 0.25 mg/g, respectively.

Based on the measurements of the various leaf traits, red prickly ash had longer and wider petioles and more compound leaves than green prickly ash, whereas green prickly ash generally had larger leaves. Leaf length, width, thickness, and area were larger in green than in red prickly ash; however, the difference was not large in the case of dry leaf weight. The fresh weight of red prickly ash leaves was far less than that of green prickly ash leaves; the water content of red prickly ash leaves was lower than that of green prickly ash leaves. The photosynthetic pigment content of red prickly ash was higher than that of green prickly ash, and the chlorophyll a, chlorophyll b, total chlorophyll, and carotenoid contents were higher in red prickly ash than in green prickly ash.

### Principal component analysis of prickly ash leaf traits

#### Principal component analysis

Principal component analysis (Tables 4, 5) was performed on the 14 leaf traits of prickly ash. Three principal components were obtained according to the principle of eigenvalues greater than or equal to one or a cumulative contribution rate greater than 85%. The eigenvalues for the first three principal components were 7.09, 3.41, and 1.79, respectively, providing a cumulative contribution rate of 87.78%. Therefore, these three principal components were considered to represent the main characteristic information of the 14 leaf traits of the 37 prickly ash germplasms from 18 different origins.

**Table 4:** Characteristic value, contribution rate, and the cumulative contribution rate of principal component analysis of prickly ash leaf traits.

Main component	Eigenvalue	Contribution rate/%	Cumulative contribution rate/%
1	7.09	50.63	50.63
2	3.41	24.34	74.97
3	1.79	12.81	87.78
4	0.58	4.13	91.91
5	0.44	3.17	95.09
6	0.28	2.00	97.08
7	0.16	1.14	98.22
8	0.10	0.75	98.97
9	0.05	0.39	99.36
10	0.04	0.32	99.68
11	0.03	0.20	99.88
12	0.02	0.11	99.99
13	0.00	0.01	100.00
14	0.00	0.00	100.00

**Table 5:** Principal component analysis of the first three principal components of 14 leaf traits.

Trait	Principle component		
	1	2	3
Petiole length	− 0.46	− 0.58	0.62
Petiole width	− 0.15	− 0.75	0.32
Leaf length	0.84	0.24	0.43
Leaf width	0.79	0.30	0.41
Leaf thickness	0.83	0.18	− 0.31
Number of compound leaflets	− 0.75	− 0.54	0.07
Leaf area	0.91	0.22	0.22
Leaf index	0.89	0.32	− 0.10
Fresh weight	0.85	0.06	0.34
Dry weight	0.44	− 0.44	0.60
Chlorophyll a	− 0.70	0.64	0.15
Chlorophyll b	− 0.64	0.63	0.36
Total chlorophyll	− 0.70	0.65	0.20
Carotenoids	− 0.59	0.68	0.36

The contribution of the first principal component to the variation in traits was 50.63%. Comparing the absolute values of each feature vector, the first principal component was mainly affected by leaf length (0.84), leaf thickness (0.83), leaf area (0.91), leaf shape index (0.89), and fresh leaf weight (0.85), which reflected the index characteristics related to prickly ash leaf size. The contribution rate of the second principal component was 24.34%, which was mainly affected by petiole width (− 0.75), chlorophyll a (0.64), chlorophyll b (0.63), total chlorophyll (0.65), and carotenoid (0.68) contents, reflecting the characteristics of the indices related to the leaf color. The contribution of the third principal component was 12.81%, which was mainly affected by petiole length (0.62) and dry leaf weight (0.60). Based on the contribution rate of the principal components, three representative indices (leaf area (0.91), leaf type index (0.89), and fresh leaf weight (0.85)) were selected, and their eigenvectors were higher than 0.85, indicating that they were the main factors causing the differences in the leaf traits of prickly ash germplasm resources and can be used as the main indices for screening and identification of the leaf traits of prickly ash germplasm.

#### Principal component analysis of leaf traits

The comprehensive scores of the leaf traits of the 18 prickly ash germplasms were calculated and ranked (Table 6). The top nine comprehensive scores for leaf traits were related to the origin of green prickly ash. The green prickly ash leaf size and weight were larger than those of red prickly ash. Therefore, the score for green prickly ash in the comprehensive evaluation of leaf traits was generally higher, ranking first. The results of the principal component factor score ranking were consistent with those of the cluster analysis. The prickly ash germplasm resources could be divided into two categories by using the leaf characteristics: the green prickly ash with the highest score and the red prickly ash with the lowest score.

**Table 6:** Principal component factor scores and ranking table.

Origin	F_1_	F_2_	F_3_	Synthesis F scores	Ranking
Hancheng, Shanxi	− 1.58	− 1.30	− 0.79	− 1.38	13
Tongchuan, Shanxi	− 3.74	0.34	1.04	− 1.91	15
Tianshui, Gansu	− 3.30	− 0.89	0.18	− 2.12	17
Wudu, Gansu	− 3.14	− 0.55	0.56	− 1.88	14
Yuncheng, Shanxi	− 3.48	− 1.14	0.46	− 2.25	18
Jiaozuo, Henan	− 3.30	− 0.68	0.84	− 1.97	16
Asakura, Japan	− 1.16	0.25	0.41	− 0.54	12
Yaan, Sichuan	0.20	− 0.28	− 2.00	− 0.25	11
Yingshan, Sichuan	1.16	0.13	− 1.94	0.42	9
Hongya, Sichuan	2.02	0.08	− 1.37	0.99	6
Zunyi, Guizhou	− 0.89	1.63	− 0.43	− 0.13	10
Rongchang, Chongqing	2.36	2.78	0.85	2.26	2
Yongchuan, Chongqing	1.82	4.44	1.97	2.57	1
Jiangjin, Chongqing	1.09	1.38	− 1.8	0.75	7
Kunming, Yunnan	3.61	− 1.87	0.16	1.59	4
Yongshan, Yunnan	0.82	1.25	− 0.98	0.68	8
Honghe, Yunnan	4.09	− 2.34	2.94	2.14	3
Qujing, Yunnan	3.43	− 3.26	− 0.1	1.06	5

## Materials and methods

### General details of test site

The experiment was conducted in the prickly ash test base of the Chongqing University of Arts and Sciences in 2022. The tested germplasm resources were all prickly ash plants that had entered a high-yield period for more than 3 years. The test area has a subtropical monsoon humid climate, with an average annual temperature of 17.7 °C, average annual precipitation of 1015 mm, average annual sunshine of 1218.7 h, and an average annual frost-free period of 317 days. The tested soil was purple. The basic fertility of the 0–30 cm soil layer was as follows: available nitrogen was 36.51 mg/kg; available phosphorus was 105.33 mg/kg; available potassium was 265.72 mg/kg; total nitrogen was 1.13 g/kg; total phosphorus was 15.72 g/kg; total potassium was 1.83 g/kg; the organic matter was 16.21 g/kg. The pH was 7.01.

### Experimental materials

The experimental materials included 37 prickly ash germplasm resources from 18 different origins at the test base. The origins of each prickly ash germplasm and its ecological and geographical factors are listed in Table 7. The experimental research on the plants described in this study comply with institutional, national and international guidelines.

**Table 7:** List of plant materials and details.

No	Variety	Origin	Minimum temperature (°C)	Average elevation (m)	Longitude	Latitude	Fruit color
1	NQ1H	Hancheng, Shanxi, China	− 17	1050	110.44°E	35.48°N	Red
2	NQ2H	Hancheng, Shanxi, China	− 17	1050	110.44°E	35.48°N	Red
3	CJ	Hancheng, Shanxi, China	− 17	1050	110.44°E	35.48°N	Red
4	WCDHP	Hancheng, Shanxi, China	− 17	1050	110.44°E	35.48°N	Red
5	HGHJ	Hancheng, Shanxi, China	− 17	1050	110.44°E	35.48°N	Red
6	PTJ	Tongchuan, Shanxi, China	− 15	1120	108.95°E	34.90°N	Red
7	SZT	Tongchuan, Shanxi, China	− 15	1120	108.95°E	34.90°N	Red
8	TSWC	Tianshui, Gansu, China	− 12	1550	105.90°E	34.57°N	Red
9	JQWC	Wudu, Gansu, China	− 8	2100	104.92°E	33.40°N	Red
10	BYH	Yuncheng, Shanxi, China	− 15	375	111.57°E	35.49°N	Red
11	JYH	Jiaozuo, Henan, China	− 11	700	113.40°E	35.10°N	Red
12	RBHJ	Asakura, Japan	− 5	654	130.67°E	33.42°N	Red
13	CCSJ	Asakura, Japan	− 5	654	130.67°E	33.42°N	Red
14	GJ	Yaan, Sichuan, China	− 2	3152	103.06°E	30.03°N	Red
15	NLJ	Yaan, Sichuan, China	− 2	3152	103.06°E	30.03°N	Red
16	XJ	Yaan, Sichuan, China	− 2	3152	103.06°E	30.03°N	Green
17	MLJ	Yingshan, Sichuan, China	− 2	1330	106.57°E	31.08°N	Green
18	TJ	Hongya, Sichuan, China	− 1	1753	103.37°E	29.92°N	Green
19	YHJ	Zunyi, Guizhou, China	− 4	1050	106.95°E	27.66°N	Red
20	RCWC	Rongchang, Chongqing, China	− 1	350	105.61°E	29.42°N	Green
21	WCFZ	Yongchuan, Chongqing, China	− 1	300	105.93°E	29.36°N	Green
22	WCZP	Yongchuan, Chongqing, China	− 1	300	105.93°E	29.36°N	Green
23	XYTJ	Jiangjin, Chongqing, China	0	1100	106.26°E	29.32°N	Green
24	TZJ	Jiangjin, Chongqing, China	0	1100	106.26°E	29.32°N	Green
25	JYQ	Jiangjin, Chongqing, China	0	1100	106.26°E	29.32°N	Green
26	SJ	Jiangjin, Chongqing, China	0	1100	106.26°E	29.32°N	Green
27	EWJ	Jiangjin, Chongqing, China	0	1100	106.26°E	29.32°N	Green
28	YL1H	Kunming, Yunnan, China	− 4	2150	102.83°E	24.88°N	Green
29	YL2H	Kunming, Yunnan, China	− 4	2150	102.83°E	24.88°N	Green
30	YQ1H	Yongshan, Yunnan, China	− 3	1770	103.63°E	28.24°N	Green
31	YQ2H	Yongshan, Yunnan, China	− 3	1770	103.63°E	28.24°N	Green
32	HHHJ	Yongshan, Yunnan, China	− 3	1770	103.63°E	28.24°N	Green
33	LFJ	Yongshan, Yunnan, China	− 3	1770	103.63°E	28.24°N	Green
34	HNJ	Honghe, Yunnan, China	0	1500	102.42°E	23.37°N	Green
35	DYSJ	Honghe, Yunnan, China	0	1500	102.42°E	23.37°N	Green
36	HYXJ	Qujing, Yunnan, China	− 3	2356	103.79°E	25.53°N	Green
37	SCHHJ	Qujing, Yunnan, China	− 3	2356	103.79°E	25.53°N	Green

### Test method

#### Determination of leaf traits

Before the prickly ash harvest period, 5 plants with consistent growth status and normal results were selected for sampling. The leaf trait sampling method was as follows: Leaves were collected from annual branches, and 30 leaves without mechanical damage or pests were selected from each branch. Leaf length, leaf width, leaf thickness, petiole length, petiole width, leaflet number of pinnate compound leaves, fresh leaf weight, and dry leaf weight were measured, and the leaf shape index and leaf area were calculated.

For measuring chlorophyll content, the absorbance of the chlorophyll extract at 470, 646, and 663 nm was determined using spectrophotometry. The chlorophyll a, chlorophyll b, total chlorophyll, and carotenoid contents were calculated using the Arnon formula^24^. Each treatment was repeated three times, and the average values were calculated.

### Data sources of eco-geographical factors

The annual minimum temperature of the origin, measured by the China Meteorological Collection Network and the meteorological stations of the origin, was recorded. Altitude, longitude, and latitude were determined for each origin.

### Data statistics and analysis

The physiological and biochemical index data of different prickly ash germplasm resources from different origins were organized. Statistical data analysis was performed using Microsoft Excel 2019 and SPSS 19.0 (IBM Corp. Released 2010. IBM SPSS Statistics for Windows, Version 19.0. Armonk, NY: IBM Corp.).

### Principal component analysis

First, KMO and Bartlett's sphericity tests were performed on the standardized data to examine the suitability of principal component extraction. The eigenvalues and variance contribution rates of each component were then analyzed. Finally, the variance contribution rate corresponding to the extracted principal components was used as the weight, and the weighted sum method was used to calculate the comprehensive scores of the spike traits of different prickly ash germplasms. The formula is as follows:

$${\text{F}} = {\text{A}}_{1} {\text{F}}_{1} + {\text{A}}_{2} {\text{F}}_{2} + {\text{A}}_{3} {\text{F}}_{3} , \ldots + {\text{A}}_{n} {\text{F}}_{n}$$

where F is the comprehensive score, Fn is the score of the nth principal component, and An is the variance contribution rate of the nth principal component.

The principal component eigenvector coefficient is calculated as follows:

$${\text{Eigenvector}} \, {\text{coefficients}} \, {\text{of}} \, {\text{principal}} \, \text{components} = \frac{{\text{Principal}} \, {\text{component}} \, {\text{load}} \, {\text{value}}}{{\text{Corresponding}} \, {\text{principal}} \, {\text{component}} \, {\text{eigenvalues}}}$$

After calculating the eigenvector coefficients of each principal component, the score function expressions for the first three principal components were constructed as follows:

$$\begin{aligned} {\text{F}}_{{1}} = & - {0}{\text{.17X}}_{{1}} - {0}{\text{.06X}}_{{2}} + {0}{\text{.32X}}_{{3}} + {0}{\text{.30X}}_{{4}} + {0}{\text{.31X}}_{{5}} - {0}{\text{.28X}}_{{6}} + {0}{\text{.34X}}_{{7}} + {0}{\text{.33X}}_{{8}} \\ & + {0}{\text{.32X}}_{{9}} + {0}{\text{.17X}}_{{{10}}} - {0}{\text{.26X}}_{{{11}}} - {0}{\text{.24X}}_{{{12}}} - {0}{\text{.26X}}_{{{13}}} - {0}{\text{.22X}}_{{{14}}} \\ \end{aligned}$$

$$\begin{aligned} {\text{F}}_{{2}} = & - {0}{\text{.31X}}_{{1}} - {0}{\text{.41X}}_{{2}} + {0}{\text{.13X}}_{{3}} + {0}{\text{.16X}}_{{4}} + {0}{\text{.10X}}_{{5}} - {0}{\text{.29X}}_{{6}} + {0}{\text{.12X}}_{{7}} + {0}{\text{.17X}}_{{8}} \\ & + {0}{\text{.03X}}_{{9}} - {0}{\text{.24X}}_{{{10}}} + {0}{\text{.35X}}_{{{11}}} + {0}{\text{.34X}}_{{{12}}} + {0}{\text{.35X}}_{{{13}}} + {0}{\text{.37X}}_{{{14}}} \\ \end{aligned}$$

$$\begin{aligned} {\text{F}}_{{3}} = & {0}{\text{.46X}}_{{1}} + {0}{\text{.24X}}_{{2}} + {0}{\text{.32X}}_{{3}} + {0}{\text{.30X}}_{{4}} - {0}{\text{.24X}}_{{5}} + {0}{\text{.05X}}_{{6}} + {0}{\text{.16X}}_{{7}} - {0}{\text{.07X}}_{{8}} \\ & + {0}{\text{.25X}}_{{9}} + {0}{\text{.45X}}_{{{10}}} + {0}{\text{.11X}}_{{{11}}} + {0}{\text{.27X}}_{{{12}}} + {0}{\text{.15X}}_{{{13}}} - {0}{\text{.27X}}_{{{14}}} \\ \end{aligned}$$

where F_1_, F_2_, and F_3_ represent the feature vector weight values of the first to third principal components, respectively, and X_1_, X_2_, X_3_,…X_14_ represent the petiole length, petiole width, leaf length, leaf width, leaf thickness, leaflet number, leaf area, leaf type index, fresh leaf weight, dry leaf weight, and chlorophyll a, chlorophyll b, total chlorophyll, and carotenoid contents of each prickly ash germplasm after standardization, respectively.

The comprehensive score of prickly ash leaf traits was calculated as follows:

$${\text{F}} = \frac{{{\text{a}}_{1} }}{{{\text{a}}_{1} + {\text{a}}_{2} + {\text{a}}_{3} }}{\text{F}}_{1} + \frac{{{\text{a}}_{1} }}{{{\text{a}}_{1} + {\text{a}}_{2} + {\text{a}}_{3} }}{\text{F}}_{2} \frac{{{\text{a}}_{1} }}{{{\text{a}}_{1} + {\text{a}}_{2} + {\text{a}}_{3} }}{\text{F}}_{3}$$

where F is the comprehensive score of the prickly ash leaf traits, and a_1_, a_2_, and a_3_ represent the contribution rates of the first to third principal components, respectively. Substituting the data into the above formula, the comprehensive score for prickly ash leaf traits is calculated as follows:

$${\text{F}} = {0}{\text{.58F}}_{{1}} + {0}{\text{.28F}}_{{2}} + {0}{\text{.15F}}_{{3}}$$

Finally, the standardized data of each trait were substituted into the F_1_–F_5_ functions; that is, the principal component factor scores for F_1_–F_5_ were obtained and substituted into the F function.

### Statistics and analysis

The data were analyzed using one-way analysis of variance (ANOVA) using the least significant difference test with three replicates for each treatment combination using SPSS 20.0 (IBM Corp. Released 2011. IBM SPSS Statistics for Windows, Version 20.0. Armonk, NY: IBM Corp.). The means were tested using the least significant difference at p = 0.05 (LSD ≤ 0.05).

### Ethics approval and consent to participate

The plant materials used in this study were collected from the experimental field of *Zanthoxylum bungeanum* at the College of Landscape Architecture and Life Sciences, Chongqing University of Arts and Sciences. Field permission was not necessary to collect the plant samples for this study. The authors declare that experimental research on the plants described in this study complies with institutional, national, and international guidelines.

## Discussion

As an organ that enables direct communication between plants and their environment, leaves are sensitive to changes in eco-geographical factors; therefore, leaf traits usually directly reflect the adaptability of plants to the environment^11,25^. Luo et al. observed that the coefficient of variation of the narrow wing width of leaves among prickly ash germplasm resources was the largest^26^. The results of the present study showed that the coefficient of variation of the 14 leaf traits of prickly ash germplasm resources was higher than 10%, indicating that the genetic variation in leaf traits among prickly ash germplasm resources is abundant. The coefficient of variation of the compound leaflet number was 49.86%, which was the highest among all leaf traits, followed by that of chlorophyll b content and petiole length at 28.31% and 27.99%, respectively. The coefficient of variation for leaf thickness, which was the lowest, stood at 11.37%.

A cluster analysis of prickly ash germplasm resources of different origins was performed using 14 leaf traits. The results showed that the 37 prickly ash germplasm resources from the 18 origins could be divided into 2 groups at 15 Euclidean distances. The first category included 17 prickly ash germplasms from 9 origins, including Hancheng in Shaanxi, Tongchuan in Shaanxi, and Tianshui in Gansu. Except for Xiaojiao, all these samples were from red prickly ash. They are characterized by small leaves, long and wide petioles, and more compound leaves. The second category included 20 prickly ash germplasms from 9 origins, including Yingshan in Sichuan, Hongya in Sichuan, and Rongchang in Chongqing. These samples were all from green prickly ash, which had larger leaves than red prickly ash, and their leaf length, width, thickness, area, and fresh weight were larger than those of red prickly ash. Three traits–leaf area, leaf type index, and leaf fresh weight–were screened from fourteen leaf traits using principal component analysis, which we observed could be used as the main indices for screening and identifying leaf traits of prickly ash germplasm. Furthermore, 37 prickly ash germplasms from 18 origins were comprehensively evaluated for leaf traits. The results showed that the origin scores of the prickly ash germplasm resources were higher and ranked at the top, and those of the prickly ash germplasm resources were lower and ranked at the bottom, which was consistent with the results of the cluster analysis.

**Figure 3:**
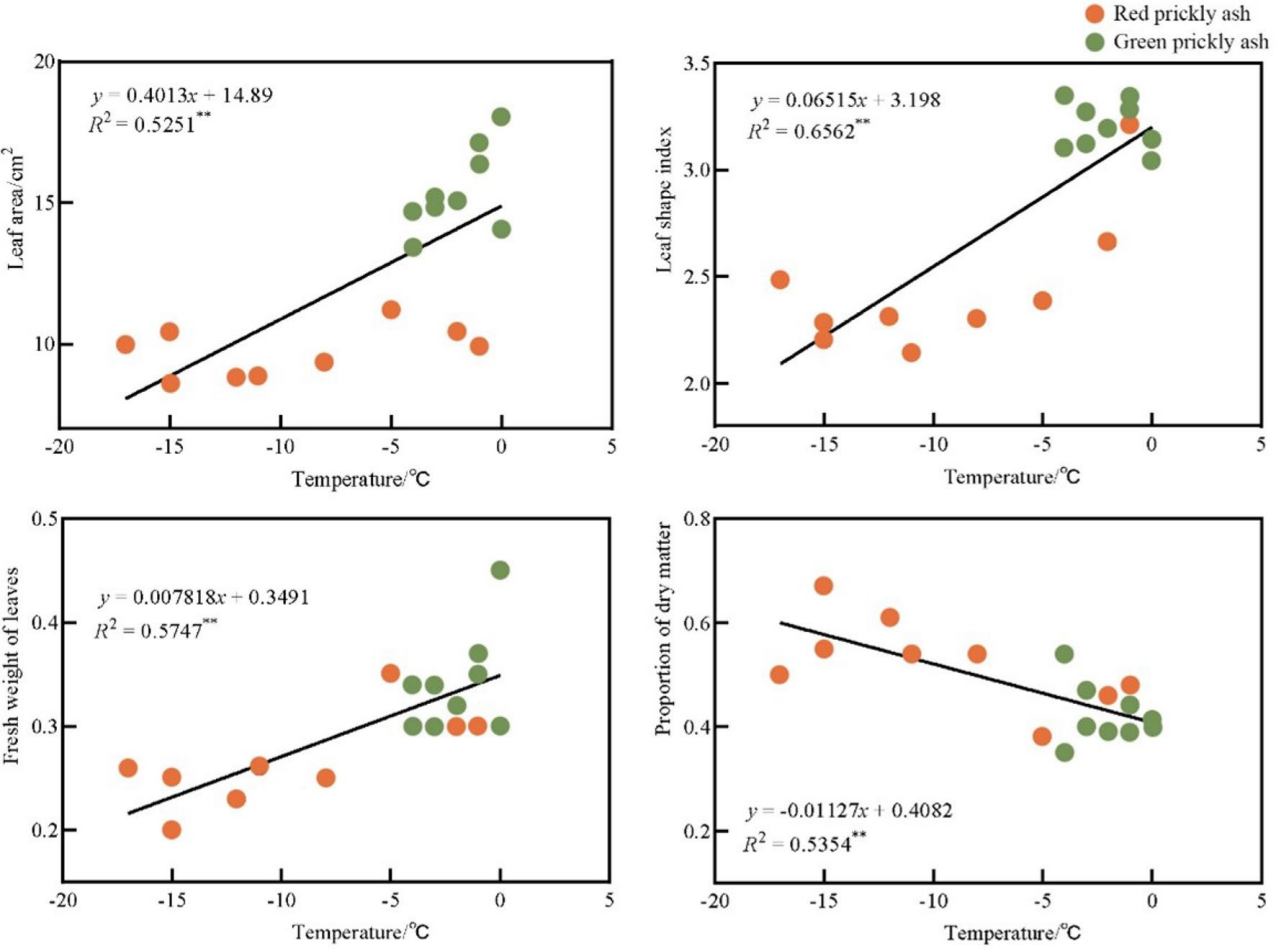
Correlation analysis between the temperature of origin and leaf traits of prickly ash. “**” indicates an extremely significant difference at *p* < 0.01.

Temperature is a key climatic factor affecting the geographical distribution of plants^27,28^. The leaves of the same plant species also change when they experience different temperature environments for a long time. The leaf width of Quercus variabilis forests is significantly negatively correlated with annual average temperature, and the leaf width directly affects the leaf area; therefore, the germplasm originating from low-temperature areas has smaller leaves^29^. In addition, the leaf size of plants is significantly and positively correlated with the annual average temperature, and the leaf area of plants significantly increases after short-term warming treatment^30,31^. The results of this study showed that the temperature of the origin significantly correlated with the leaf area (*R*^2^ = 0.5251 **), leaf type index (*R*^2^ = 0.6562 **), leaf fresh weight (*R*^2^ = 0.5747 **), and leaf dry fresh ratio (*R*^2^ = 0.5354 **) of the prickly ash germplasm (Fig. 3). The leaves of the prickly ash germplasm originating from lower-temperature areas were smaller than those originating from warmer areas, which is consistent with previous studies on other plant species^32,33^. With an increase in the temperature at the origin, the leaf area and weight of the plants increased. The results of the further analysis showed that the annual minimum temperature at the origin of green prickly ash was above –5 °C, whereas most of the red prickly ash originated from areas where this annual minimum was below − 5 °C, which again indicated that the temperature differences between the origins of green and red prickly might be an important reason for their difference in leaf traits.

**Figure 4:**
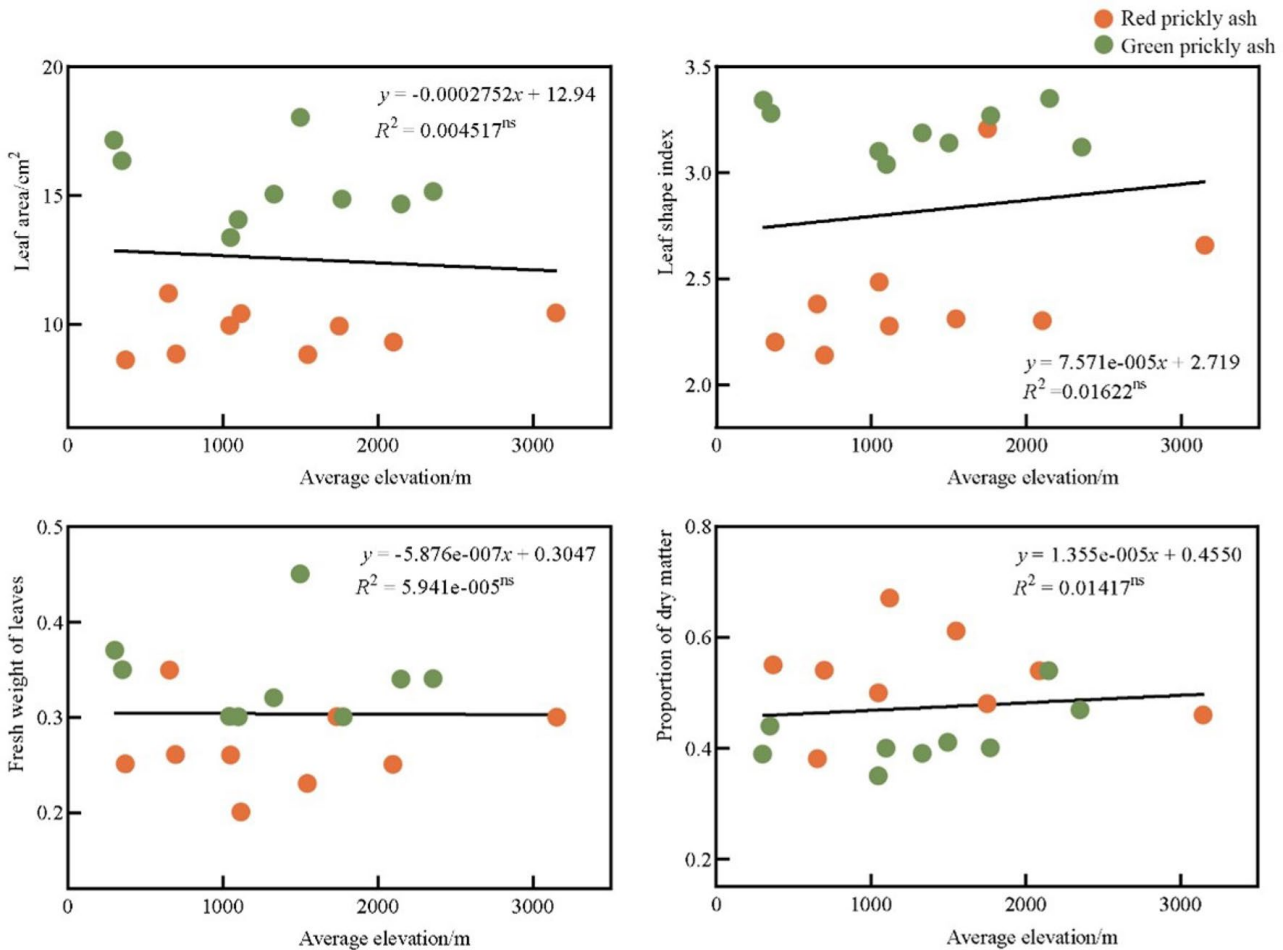
Correlation analysis between altitude and leaf traits of prickly ash. *ns* indicates that the difference or relationship being tested is not statistically significant.

As an important eco-geographical factor, location, and origin, altitude also strongly impacts the leaf traits of plants^34^. McDonald et al. noted that the leaf area of plants significantly decreased with increasing altitude^35^. Li et al. also observed that the leaf area of four desert shrubs significantly decreased with an increase in altitude and that altitude negatively correlated with the specific leaf area of plants^36^. Altitude is the most important environmental variable affecting variations in plant functional traits. The specific leaf area and volume of plants are significantly negatively correlated with increasing altitude^37^. The results of this study showed that the altitude of origin was not significantly correlated with the leaf area, leaf shape index, or leaf weight of the prickly ash germplasm (Fig. 4), which is inconsistent with the results of previous studies. This could have been due to the large difference in latitude between the origins considered in this study, and the latitude had a stronger impact on climatic factors such as temperature than altitude; therefore, the correlation between leaf traits and altitude of the prickly ash germplasm was insignificant^38^. The results of further analysis showed that at similar latitudes, altitude also had an important impact on the leaf traits of prickly ash germplasm. For example, in Chongqing, Yongchuan, and Chongqing, Jiangjin (29.36°N and 29.32°N, respectively), the latitudes do not differ much, but their altitudes differ (300 m and 1100 m). Significant differences were identified in leaf traits such as length (8.59 cm and 6.68 cm, respectively), width (3.02 cm and 2.38 cm, respectively), and fresh weight (0.37 g and 0.30 g, respectively). The leaves of the prickly ash germplasm originating from high-altitude areas were smaller than those from low-altitude areas, which is consistent with previous studies.

**Figure 5:**
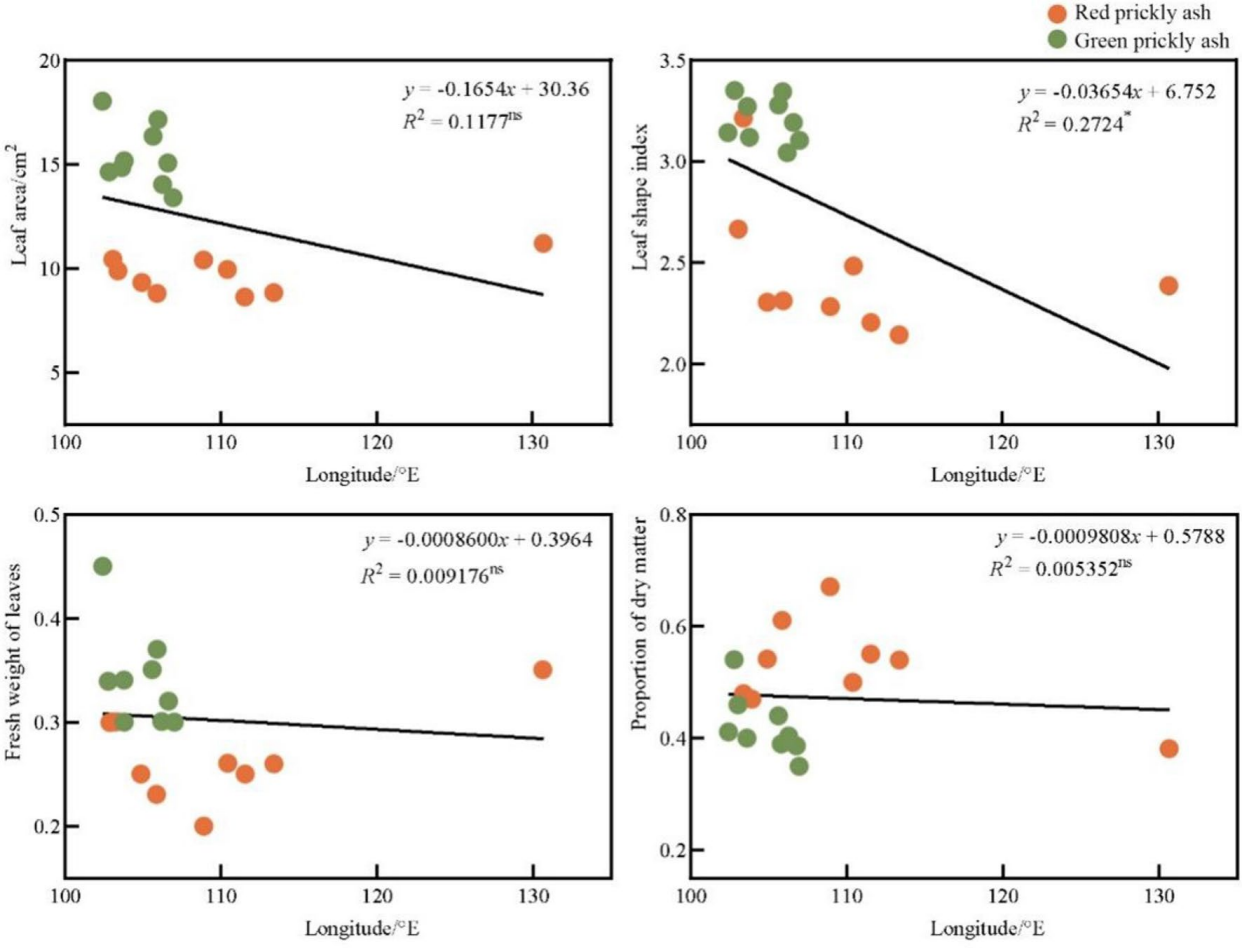
Correlation analysis between the longitude of various origins and leaf traits of prickly ash. “*” indicates a significant difference at *p* < 0.05, ns indicates that the difference or relationship being tested is not statistically significant.

**Figure 6:**
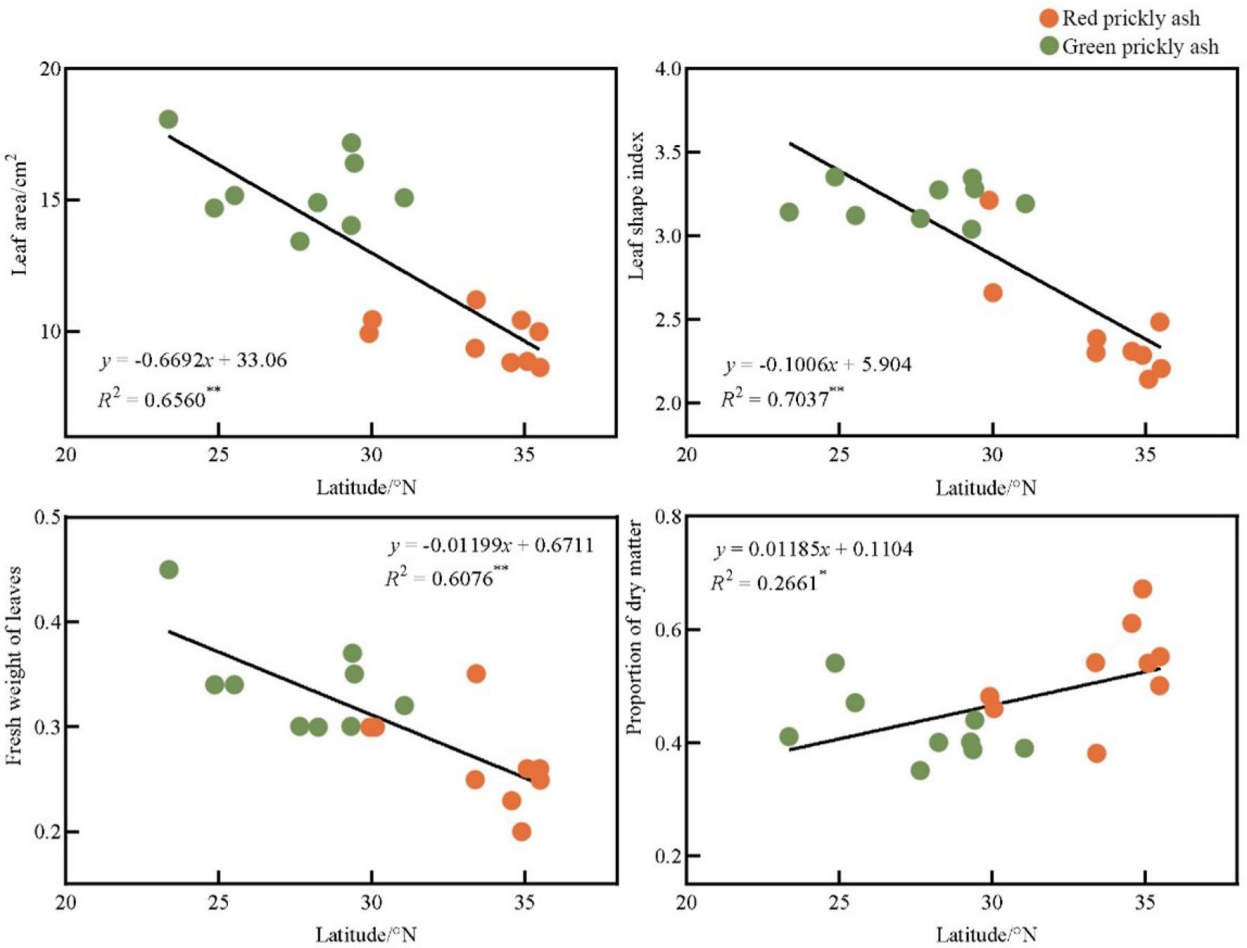
Correlation analysis between the latitude of various origins and leaf traits of prickly ash. “*”Indicates a significant difference at *p* < 0.05, “**”indicates an extremely significant difference at *p* < 0.01.

An inseparable relationship exists between the latitude, longitude, and temperature of a location. The latitude directly affects the temperature, which in turn affects the leaf traits of the plant. Wang reported a significant correlation between the leaf area of plants and changes in latitude^39^. The leaf area index of plants at low latitudes was usually higher, whereas, at middle and high latitudes, the leaf area index gradually decreased with increasing latitude. Ou also reported that latitude was significantly correlated with plant leaf area^40^. Phoebe zhennan leaves were observed to gradually become rounder from west to east and narrower and longer from south to north. Xiao et al. reported similar results for camphor tree leaves^41^. Latitude was negatively correlated with leaf area, whereas morphological factors were negatively correlated with longitude. The results of this study showed that no significant correlation exists between the longitude of the origin, leaf area, and leaf weight of prickly ash, but a significant correlation was observed with the leaf type index (*R*^2^ = 0.2724 *) (Fig. 5). The latitude of the variety origin was significantly negatively correlated with the leaf area (*R*^2^ = 0.6560 **), leaf type index (*R*^2^ = 0.7037 **), and fresh leaf weight (*R*^2^ = 0.6076 **) of prickly ash (Fig. 6), which was consistent with previous research results. The latitude had a stronger correlation with the leaf traits of prickly ash compared to longitude, indicating that latitude was the main factor causing the differences in leaf traits of the prickly ash germplasm. With an increase in the latitude of the origin, the leaves of the prickly ash germplasm gradually became smaller, the leaf weight gradually became lighter, and the leaf shape index gradually decreased, which is consistent with previous research results.

## Conclusions

The leaf traits of prickly ash germplasms of different origins were significantly different, showing a rich diversity of leaf traits. The coefficients of variation for the 14 leaves were above 10%. The coefficient of variation of the compound leaflet number was the highest (49.86%) among all considered leaf traits, and the coefficient of variation of leaf thickness was the lowest (11.37%). The results of the cluster analysis showed that the 37 germplasm resources of prickly ash from 18 origins could be divided into 2 groups at a Euclidean distance of 15 using leaf traits. The first category included 17 prickly ash germplasm lines from 9 origins, including Hancheng in Shaanxi, Tongchuan in Shaanxi, and Tianshui in Gansu. Except for Xiaojiao, all samples contained red prickly ash. Their leaves are characterized by small leaves, long and wide petioles, and more compound leaves. The second category included 20 prickly ash germplasms from 9 origins, including Yingshan in Sichuan, Hongya in Sichuan, and Rongchang in Chongqing. All were green prickly ash, the leaves of which were larger than those in the first category. Leaf length, leaf width, leaf thickness, leaf area, leaf fresh weight, and the number of compound leaves were larger than those in the first category. Principal component analysis of the 14 leaf traits was used to screen leaf area, leaf type index, and leaf fresh weight. The main factors causing the differences in the leaf traits of prickly ash germplasm resources can be used as the main indices for the screening and identification of leaf traits in prickly ash germplasm. The main indices selected were used to comprehensively evaluate the germplasm of prickly ash. The results of the evaluation showed that the origin scores of green prickly ash germplasm resources were higher and ranked first, and the origin scores of red prickly ash germplasm resources were lower and ranked second, which was consistent with the results of the cluster analysis. Among the ecological and geographical factors of the origins, the latitude of the origin had the strongest correlation with the leaf traits of the prickly ash germplasm. With an increase in latitude, the leaves of prickly ash gradually became smaller, leaf weight gradually decreased, and the leaf shape index gradually decreased. The temperature was the next most influential factor. The leaves of the prickly ash germplasm from the warm-climate areas were larger and heavier than those of cold-climate regions. Altitude and longitude had no significant effects on the leaf traits of the prickly ash germplasm. However, at similar latitudes, the leaves of the prickly ash germplasm in high-altitude areas were smaller, and the leaves of the prickly ash germplasm in low-altitude areas were larger. In addition, the changes of morphological and physiological indexes of leaves are determined by ecological and geographical factors, but the specific mechanism of action has not been fully understood. In the future, further research can be carried out in order to deepen the understanding of the relationship between leaf traits and ecological geographical factors.

## Data Availability

All data supporting the findings of this study have been included in this article.
